# Person-Centered Care for Family Caregivers: Evaluating an Education Program for the Healthcare Workforce

**DOI:** 10.1093/geroni/igab046.1884

**Published:** 2021-12-17

**Authors:** Sharon Anderson, Jasneet Parmar, Cheryl Pollard, Bonnie Dobbs, Myles Leslie, Gwen McGhan

**Affiliations:** 1 Faculty of Medicine & Dentistry University of Alberta, Edmonton, Alberta, Canada; 2 Department of Family Medicine, University of Alberta, Edmonton, Alberta, Canada; 3 McEwan University, Edmonton, Alberta, Canada; 4 University of Alberta, Edmonton, Alberta, Canada; 5 University of Calgary, Calgary, Alberta, Canada

## Abstract

Background: While family caregivers [FCGs] provide 75-90% of care for people living in the community, most healthcare providers are not trained to provide person-centered care to FCGs. We followed research recommendations that the healthcare workforce receive competency-based education to identify, assess, support and partner with FCGs. Objective: Mixed methods evaluation healthcare workforce education program. Approach: We began by coining the concept “caregiver-centered care,” defining it as a collaborative working relationship between families and healthcare providers aimed at person-centered support for FCGs. From this definition, interdisciplinary stakeholders including FCGs (n=101) co-designed the Foundational Caregiver-Centered Care education. Learning resources included six competency-aligned educational modules with videos and interactive exercises that encourage reflection. Kirkpatrick Barr’s healthcare training evaluation framework underpinned our mixed methods evaluation. We measured participant’s reaction to the education (Level 1) and changes in learner’s knowledge and confidence to work with FCGs (Level 2). Results: 352 healthcare providers completed the education online (caregivercare.ca). Learners were satisfied with quality of education (Mean 4.75/5; SD=.5) and the education increased their motivation to learn more about caregiver-centered care (Mean 4.75/5; SD .5). Student’s paired samples T-test indicates pre-post education changes in knowledge and confidence to work with FCGs were significant [Pre (M=37.8, Sd=7.6) to post (M=47.2, SD=3.5) t (125) = -14.39, p<.0005 (two-tailed)]. Qualitative results derived from open responses mirrored the quantitative results. Conclusion: The Caregiver-Centered Care education provides a foundation for educating healthcare providers working with FCGs to provide care to FCGs to maintain their wellbeing and sustain care.

